# Necroptosis in Cholangiocarcinoma

**DOI:** 10.3390/cells9040982

**Published:** 2020-04-15

**Authors:** Samantha Sarcognato, Iris E. M. de Jong, Luca Fabris, Massimiliano Cadamuro, Maria Guido

**Affiliations:** 1 Department of Pathology, Azienda ULSS2 Marca Trevigiana, 31100 Treviso, Italy; 2 Department of Surgery, Section of Hepatobiliary Surgery and Liver Transplantation, University Medical Center Groningen, 9700 Groningen, The Netherlands; 3 Department of Molecular Medicine—DMM, University of Padova, 35121 Padova, Italy; 4 Department of Medicine—DIMED, University of Padova, 35121 Padova, Italy

**Keywords:** necroptosis, cholangiocarcinoma, cell death, regulated cell death

## Abstract

Necroptosis is a type of regulated cell death that is increasingly being recognized as a relevant pathway in different pathological conditions. Necroptosis can occur in response to multiple stimuli, is triggered by the activation of death receptors, and is regulated by receptor-interacting protein kinases 1 and 3 and mixed-lineage kinase domain-like, which form a regulatory complex called the necrosome. Accumulating evidence suggests that necroptosis plays a complex role in cancer, which is likely context-dependent and can vary among different types of neoplasms. Necroptosis serves as an alternative mode of programmed cell death overcoming apoptosis and, as a pro-inflammatory death type, it may inhibit tumor progression by releasing damage-associated molecular patterns to elicit robust cross-priming of anti-tumor CD8+ T cells. The development of therapeutic strategies triggering necroptosis shows great potential for anti-cancer therapy. In this review, we summarize the current knowledge on necroptosis and its role in liver biliary neoplasms, underlying the potential of targeting necroptosis components for cancer treatment.

## 1. Introduction

A continuous process of cell death (CD) and renewal takes place on a daily basis in all mammal organ systems. Disturbances in these processes interrupt the normal regulation of development and tissue homeostasis with the potential to induce pathological conditions, including cancer [1]. Therefore, a better understanding of how balance between CD and survival is controlled is highly relevant in many fields, from developmental alterations to human diseases and cancer research, and may facilitate the development of novel effective therapies.

Necroptosis is a type of tightly regulated cell death (RCD) mimicking the morphological features of necrosis [2]. Similar to non-regulated necrosis, it represents an inflammatory mode of CD [2]. Necroptosis and its molecular players contribute to embryonic and post-natal development and participate in tissue homeostasis [2]. Several studies on cell lines, animal models, and human tissue have been conducted over the last ten years, demonstrating the involvement of necroptosis in the pathogenesis and natural course of different pathological conditions. In addition to its key role in inflammatory conditions, necroptosis seems to be involved in the regulation of cancer biology, including tumorigenesis, metastasis development, and cancer immunity [3].

A plethora of evidence has shown that a switch from one type of CD to another is possible and regulated by specific molecules. In particular, the inhibition of caspase-8 shifts extrinsic apoptosis to a necrosis type of CD, due to the activation of receptor-interacting serine/threonine-protein kinase 3 (RIPK3) and mixed lineage kinase domain-like (MLKL) [4]. Therefore, necroptosis is an alternative mode of CD when the caspase-8-dependent apoptotic pathway is blocked. It is now well-established that various stimuli can initiate necroptosis, including intra- and extracellular factors, such as tumor necrosis factor α (TNFα) and reactive oxygen species (ROS) [5].

A hallmark of cancer is the ability of malignant cells to evade apoptosis. Therefore, the induction of necroptosis could be an alternative strategy for killing cancer cells. Several therapeutics able to affect the necroptotic cascade have recently been developed, and a few of them are already in phase 1 trials for the treatment of inflammatory diseases. Moreover, necroptosis modulation is becoming the target of several new anti-cancer strategies. In fact, it has been demonstrated that apoptosis-resistant tumors respond to necroptosis, and that necroptosis can create an immunogenic microenvironment that enhances tumor clearance [6]. These aspects will be further discussed in the following chapters.

Herein, we will discuss the general aspects of necroptosis and what is currently known on its involvement in cholangiocarcinoma (CCA), in order to provide perspectives for future studies in this relatively new field.

## 2. Overview on Types of Cell Death

In 2005, the Nomenclature Committee on Cell Death (NCCD) defined the first CD classification purely based on morphological criteria. This classification included three major forms of CD: types I, II, and III [7]. Type I CD, termed apoptosis, exhibits cell shrinkage, membrane blebbing, DNA fragmentation, chromatin condensation, and the formation of apoptotic bodies. Apoptosis is a form of RCD and responds to two different death signals: damage from inside the cell, such as DNA damage, which elicits an intrinsic pathway, and extracellular stimuli, such as TNFα and the Fas ligand, which are followed by an extrinsic pathway. Both pathways are well-regulated, take nearly a day to be effected, and do not involve any neighboring cell or immune cell [8]. Type II CD is known as autophagy-dependent CD and is also considered to be a form of RCD. It relies on the autophagic machinery, which usually has cytoprotective effects, leading to cell adaptation to stress (such as starvation or hypoxia). However, in the case of persistent stress stimuli, autophagy can result in cell demise. This type of CD is morphologically characterized by extensive cytoplasmic vacuolization and lysosomal degradation [8,9]. Type III CD, referred to as necrosis, is a consequence of an overwhelming cytotoxic insult, which cannot be controlled or survived by the cell. Necrosis is generally recognized as accidental CD and manifests with vacuolation of the cytoplasm, cell swelling, and membrane rupture. In contrast to apoptosis, necrosis is a fast form of CD, and its presence induces an immunogenic reaction due to the release of damage-associated molecular patterns (DAMPs), including high-mobility group box 1 and heat shock protein 70, which act as danger signals alerting the organism about a potential threat [9,10,11].

During the last decade, research in this field has taken a big leap forward, resulting in the discovery of novel triggers and mechanisms of CD. The most studied forms of CD are the three described above, but it is easy to understand how this classification system, even if still employed, became outdated and incomplete. For this reason, in 2018, the NCCD published a consensus paper with nomenclature, definitions, and an updated classification of all currently known forms of CD, centered on a molecular, biochemical, pharmacological, and functional, rather than morphological, basis, which includes a long list of all newly recognized forms of CD that has probably not yet reached its full length [9,12,13,14,15]. In this new classification, type I CD was definitively split into two different processes, termed intrinsic and extrinsic apoptosis, while type III CD was renamed mitochondrial permeability transition-driven necrosis, pointing out the importance acquired by the molecular aspects in defining CD modalities [9]. Moreover, eight new types of CD were identified and well-defined, named necroptosis, ferroptosis, pyroptosis, parthanatos, entotic CD, NETotic CD, lysosome-dependent CD, and immunogenic CD [9]. It was also clearly specified that molecular mechanisms involved in the initiation and propagation of different RCD modes exhibit a considerable degree of interconnectivity [9]. Furthermore, different from what had been reported before, the Committee stated that each type of RCD may manifest with an entire spectrum of morphological features ranging from fully necrotic to fully apoptotic, and an immunomodulatory profile ranging from anti-inflammatory and tolerogenic to pro-inflammatory and immunogenic [9].

It is now known that a specific microenvironment can elicit specialized forms of CD, some of which do not take part in normal homeostasis. Subcategories of RCD with a function in physiological conditions are necroptosis and pyroptosis, which are implicated in development and inflammation. In contrast, the initiation of other CD-related pathways (ferroptosis, parthanatos, entotic CD, NETotic CD, lysosome-dependent CD, and autophagy-dependent CD) is restricted to specific pathological circumstances or toxin exposure [15]. Interplay between these pathways has already been suggested and should be taken into consideration when exploring possible regulators or biomarkers. For example, autophagy can induce the machinery of necroptosis, which in turn involves crosstalk with apoptosis and can even stimulate parthanatos [3]. On the contrary, ferroptosis can inhibit these responses [3]. Indeed, clarifying the links between different types of CD is crucial to understanding, predicting, and eventually manipulating cell fate, particularly in pathological conditions, such as cancer.

## 3. Necroptosis

As mentioned above, necroptosis is a recently discovered type of RCD, which, in contrast to apoptosis, is characterized by morphological features of necrosis [2]. In fact, an increasingly translucent cytoplasm, the swelling of organelles, lysosomal membrane permeabilization, and an enlarged cell volume are common features of necroptosis. Nuclei remain intact with mild chromatin condensation, unlike apoptosis [16]. Necroptosis can be triggered by multiple stimuli that are sensed by specific receptors. Several intra- and extracellular signals that induce necroptosis have been described. For instance, intracellular factors such as TNFα, the Fas ligand, the tumor necrosis factor-related apoptosis-inducing ligand, interferon γ, double-stranded RNA, and adenosine triphosphate (ATP) depletion, are known to induce necroptosis, as well as extracellular events, such as the production of ROS, calcium overload, and ischemia/reperfusion injury (IRI) [5,17,18]. All these stimuli act by binding death receptors, including Fas, TNFα receptor 1 (TNFR1), and pathogen-induced receptors, such as certain toll-like receptors (TLRs) and Z-DNA binding protein 1 [5,18,19]. Whereas the downstream cascades activated by TNFR1 have been relatively well-studied, the description of other pathways is limited to a few models and publications.

**Figure 1:**
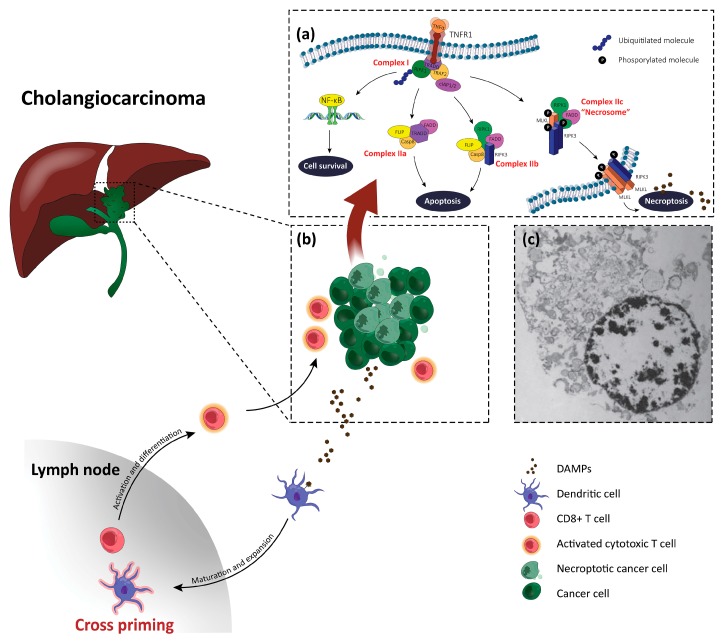
A schematic overview of necroptosis in cholangiocarcinoma (CCA). (**a**) A simplified illustration of the intracellular pathways involved in cell fate in a necroptotic CCA cell. As reported in the text, tumor necrosis factor α (TNFα) activates its receptor, TNFα receptor 1 (TNFR1), which binds a series of proteins to form complex I. The ubiquitylation of receptor-interacting serine/threonine-protein kinase 1 (RIPK1) leads to cell survival, through the nuclear factor-kappa B (NF-κB) pathway. When this process is impeded, and caspase-8 is active, complex IIa will assemble, leading to RIPK1-independent apoptosis. The deubiquitylation of RIPK1 in the presence of activated caspase-8 leads to the assembly of complex IIb and, subsequently, to RIPK1-dependent apoptosis. The inhibition of caspase-8 leads to RIPK1/receptor-interacting serine/threonine-protein kinase 3 (RIPK3)/mixed lineage kinase domain-like (MLKL) interaction, forming complex IIc, also named the necrosome. Phosphorylated MLKL and RIPK3 translocate to the plasma membrane, opening membrane pores, and resulting in damage-associated molecular patterns (DAMPs) release. (**b**) The immunogenic response to DAMPs. Dendritic cells, activated by DAMPs, travel to lymph nodes, mature and expand, and present a tumor antigens to naïve CD8+ T cells, in a process called cross-priming. T cells are then activated and differentiate into cytotoxic T cells primed to specifically attack tumor cells. (**c**) The morphology of a necroptotic cell. Translucent cytoplasm, the swelling of organelles, patches of condensed chromatin within the nucleus, an increased cell volume, and a disrupted cell membrane can be visualized by electron microscopy. Picture adopted from Vandenabeele et al. [20].

TNFα/TNFR1 binding promotes not only necroptosis, but also caspase-dependent apoptosis and activation of the nuclear factor-kappa B (NF-κB) pathway, which trigger the formation of pro-inflammatory and pro-survival complexes (Figure 1) [18,19].

TNFR1 activation leads to the formation of the so-called complex I by recruiting TNFR1-associated death domain (TRADD), TNFR-associated factor 2, RIPK1, the cellular inhibitor of apoptosis protein (cIAP) 1-2, and the linear ubiquitin chain assembly complex [18]. Upon the formation of complex I, different signaling complexes can follow, which can determine CD or survival [21]. The polyubiquitination of RIPK1 mediated by cIAP leads to the activation of the pro-survival NF-κB cascade, which, in turn, mediates the expression of genes encoding for cytoprotective molecules and stimulates cell survival [22]. However, when NF-κB or its regulators are blocked (for example, by cIAP inhibition), deubiquitylation of RIPK1 happens, which induces its release and the formation of other complexes that favor CD. Notably, the deubiquitylation of RIPK1 can also be directly promoted by drugs, such as the so-called second mitochondrial activator of caspases (Smac) mimetics [23]. The association of Fas-associated protein with the death domain (FADD), TRADD, FLICE-inhibitory protein (FLIP), and pro-caspase-8 forms cytosolic complex IIa, and the activation of pro-caspase-8 into caspase-8, promotes RIPK1-independent apoptosis [4,18]. However, in the case of knockdown of the NF-κB essential modulator or blockage of cIAPs or transforming growth factor β-activated kinase 1 (TAK1), FADD, FLIP, and pro-caspase-8 bind RIPK1 and RIPK3, forming complex IIb, which is involved in RIPK1-dependent apoptosis [18,19]. On the contrary, when caspase-8 is down-regulated or inhibited, RIPK1 interacts with RIPK3 and MLKL to form complex IIc, also called the necrosome, by which necroptosis is initiated [4,18]. Interestingly, it has also been demonstrated in cell lines (e.g., L929 cells and murine embryonic fibroblasts) and murine models (e.g., ethanol-induced liver injury mouse model) that RIPK3 may also induce necroptosis in the absence of RIPK1, but how RIPK3 might directly interact with TNFR1 signaling components remains unclear [24]. The ultimate execution step consists of MLKL phosphorylation by RIPK3, triggering MLKL oligomerization, which is indispensable for its translocation from the cytosol to the plasma membrane [18,25]. MLKL oligomers mediate plasma membrane permeabilization, by destabilizing it through a pore-forming complex or by indirectly inhibiting Ca2+ or Na+ channel functions. This leads to an increase of the intracellular osmotic pressure and the opening of membrane pores, which represents the last step and one of the hallmarks of necroptotic CD [9,18]. Bursting of the membrane results in the release of DAMPs, which act as activators and amplifiers of the inflammatory response. They include molecules which only display immune activity upon release (i.e., heat shock proteins and extracellular ATP), alarmins, and specific cytokines, such as different interleukins, which are also able to initiate an inflammatory response [18]. DAMPs are recognized by a series of receptors called “pattern recognition receptors,” such as TLRs and receptor for advanced glycation end products, which activate the innate immunity and further evoke the release of cytokines. This, in turn, induces more necrosis and triggers an inflammatory cascade [4,18]. After the binding of TNFα to its receptor, it takes a few hours until DAMPs are released [26]. In a neoplastic context, DAMPs attract and activate naïve dendritic cells at the tumor site, to get rid of tumor antigens. Activated dendritic cells then travel to the lymph nodes, mature and expand, and finally present tumor antigens to naïve CD8+ T cells. This process, termed cross-priming, leads to CD8+ T cell activation and differentiation into cytotoxic T cells primed to specifically attack tumor cells (Figure 1) [27].

Even if the mechanisms involved in the necroptotic cascade are now clearer, the role of necroptosis in cancer remains complex and difficult to understand. As Gong et al. wrote, necroptosis may be both a friend and an enemy of cancer [6]. Indeed, the initiation of necroptosis can prevent tumor development in cells where apoptosis failed. However, at the same time, as a necrotic CD modality, the inflammatory response that is created after the release of DAMPs promotes angiogenesis, cell proliferation, and metastasis [6,27,28,29,30,31] (Table 1).

The expression of RIPK1, RIPK3, and MLKL, and the double ability of necroptosis of both promoting and reducing tumor development and growth, have been studied in a variety of tumors, in order to clarify the relationship with patient prognosis [6]. A reduced expression of key molecules in the necroptotic pathway has been observed in different cancer cell lines, suggesting that these cells may evade necroptosis to survive [6]. As a consequence, a lower expression of necroptosis regulators seems to correlate with a worse prognosis in several types of carcinomas (i.e., breast, colorectal, gastric, and ovarian cancer, and head and neck and cervical squamous cell carcinoma), in acute myeloid leukemia, and in melanoma [30,42,43,44,45,46,47,48]. However, the expression of RIPK1, RIPK3, and MLKL has been found to be upregulated in some cancers (i.e., glioblastoma, pancreatic, lung, and esophageal cancer), in which a high activation of the necroptotic cascade seems to be correlated with a poorer prognosis in different studies [6,36,41,49,50,51,52,53]. The role of necroptosis in liver carcinogenesis is also still not clear. In a mouse model of TAK1 deletion in parenchymal liver cells, which induces RCD, apoptosis, but not necroptosis, promoted carcinogenesis. In these experiments, a pure activation of necroptosis was related to a suppression in inflammation, proliferation, and carcinogenesis [54,55]. Commonly used human hepatoma cell lines showed a methylation-dependent loss of RIPK3 expression with a block in the activation of the necroptotic pathway, suggesting that evasion from necroptosis is an important step in hepatocyte malignant transformation [55]. Furthermore, RIPK3 re-activation by the demethylation of its promoter can re-sensitize tumor cell lines to chemotherapy, opening a new approach for hepatocellular carcinoma (HCC) treatment [55]. Altogether, the available data indicate that the actual effect of necroptosis on cancer varies according to the location, biological features, and microenvironment of each cancer type [6]. An improved understanding of the mechanisms driving necroptosis directly implies translational applications. Biomarkers of necroptotic CD might not only identify patients at risk of cancer development, but also help to delineate tumor prognosis and predict and monitor the response to treatment [54,55].

## 4. Overview on Necroptosis in Liver Disease

In healthy conditions, almost all liver cells remain in the G0 phase of the cell cycle, with little turnover and virtually no CD. In the case of liver injuries of different etiologies, hepatocellular death occurs, and the liver starts to regenerate in order to preserve its homeostasis and metabolic functions [54,55]. However, CD in the liver occurs not only as a passive response to insults, but also as a process actively induced by the host via RCD. As in other organs, it is now evident that in the liver, besides apoptosis, necroptosis represents a highly relevant form of RCD [54,55]. However, its exact role in liver biology and pathophysiology is still not well-understood. This is mainly due to the lack of simple and specific tools for detecting the activation of the necroptotic cascade in vivo [54,55,56].

Studies based on immunohistochemistry and Western blot have shown that the expression of necroptotic markers in hepatocytes is relatively low, suggesting that other cell types, including cholangiocytes, hepatic stellate cells, Kupffer cells, and endothelial cells, may participate in normal liver functions, as well as contribute to some forms of liver disease [55,57]. A robust expression of RIPK3 and MLKL has been observed in isolated endothelial and Kupffer cells, and in liver leukocytes [57,58]. RIPK3 is usually expressed at low levels in human hepatocytes and cholangiocytes, whereas an increased expression can be induced upon caspase-8 deletion and in distinct pathophysiological conditions, such as drug-induced acute liver injury [16,54,55].

The contribution of necroptosis to liver disease seems to be context-specific and dependent on the lineage of the involved cells, the etiology, the stage of the disease, and the nature and extent of co-morbidities [59,60]. Various methods, including pharmacological inhibition and gene knockout and knockdown, have been utilized to evaluate the role of necroptosis in different diseases, mostly using murine models [2,54]. As a model of acute liver injury, several groups focused on acetaminophen-induced hepatic injury (AIHI) in mice, but the results they obtained were conflicting [2,3,18,57,58,61,62,63,64,65,66,67,68]. Indeed, the involvement of necroptosis in AIHI is still questionable, and it is still not clear whether RIPK1 or RIPK3 inhibition may have a protective role in this setting [18].

In the setting of non-alcoholic fatty liver disease (NAFLD)/non-alcoholic steatohepatitis (NASH), it was initially reported that RIPK3 might maintain white adipose tissue homeostasis and prevent glucose intolerance by regulating caspase-8 expression, thus reducing adipose tissue apoptosis and inflammation [2,57,59,69,70,71]. In a high-fat diet mouse model, RIPK1 inhibition, and the subsequent downstream necrosome inactivation, improved all the histologic features of NASH, including liver inflammation and fibrosis [72]. Some studies indicated that the effects of RIPK3 and MLKL blockage are not consistent, with RIPK3 deletion that seemed to be harmful, while MLKL deletion that seemed to be beneficial in NAFLD [2,59,69,71]. However, different roles of RIPK3 have been observed in different mouse models, with some authors reporting an amelioration of liver injury and fibrosis following RIPK3 inhibition [2,18,55,73,74]. In human NAFLD/NASH biopsy samples and serum, increased RIPK3 and MLKL expressions have been described, suggesting that necroptosis may also mediate liver injury, oxidative stress, and liver fibrosis in humans, through a positive feedback loop involving the activation of Jun N-terminal kinase [2,55,73,75]. Moreover, the serum concentrations of RIPK1 and MLKL increased with histological activity, and correlated with alanine transaminase (ALT) levels in patients with NAFLD, suggesting that a blood-based non-invasive assessment of necroptosis activity could be useful for identifying patients with NASH [72].

In alcoholic liver disease, an increased RIPK3 expression has been observed in human biopsy samples, as well as in mice after chronic ethanol exposure. RIPK3 ablation can reduce an ethanol-induced increase in serum ALT, hepatic steatosis, and inflammation [2,54,55,59,76,77,78].

Controversial data are also available on the role of necroptosis in autoimmune hepatitis (AIH) [2,18,55,79,80,81,82]. A high level of RIPK1 kinase activity and RIPK3 expression has been reported in murine AIH models and in the liver tissue of AIH patients, respectively [18,37,66,83]. However, the genetic silencing of RIPK3 in a murine model could not improve AIH [18,66]. Furthermore, an elevated MLKL expression has been reported in human AIH biopsies and murine models, and it has been proven that the disease can be driven by an MLKL-dependent pathway, which seems to be independent of RIPK3 [18,67].

RIPK3 expression has been shown by immunohistochemistry in liver biopsies from patients with hepatitis B and C virus infections, suggesting that necroptosis may also occur in viral hepatitis; however, available data on this topic are still very scant [18,84,85].

Different groups have demonstrated the involvement of necroptosis in IRI during orthotopic liver transplantation, both in murine cell lines and mouse IRI models [18]. In this setting, an increased expression of necroptosis mediators has been described, and different inhibitors have been used to block the necroptotic cascade [3,18,55,86,87,88,89,90,91,92].

The activation of the necroptotic pathway has also been described in cholangiopathies [54,93,94,95]. In mice with bile duct ligation (BDL), caspase inhibitors could only moderately alleviate liver injury, meaning that apoptosis is not the only type of CD involved in cholestasis and suggesting that, as in NAFLD/NASH, a switch to necroptosis may occur [18,96]. It has been reported by two different groups that, in a TAK1-deficient mouse model of cholestasis, hepatocytes die exclusively through caspase 8-mediated apoptosis, while cholangiocytes can undergo necroptosis, triggered by RIPK1 and RIPK3 activation [38,39]. The association of necroptosis activation with biliary cell damage and the development of jaundice suggests that the use of RIPK1 inhibitors could be effective in the treatment of biliary diseases [18,38,39]. Moreover, it has recently been reported that, in BDL mice, microRNA-21 (miR-21) expression is induced and actively involved in promoting necroptosis. In those experiments, miR-21 ablation protected against necroptosis, fibrosis, and oxidative stress [2,97]. Finally, although suggested by only a few studies, it is likely that necroptosis and other types of regulated CD may be involved in cholangiopathies related to IRI and liver allograft rejection [16].

## 5. Necroptosis and Cholangiocarcinoma

CCA constitutes a heterogeneous group of liver epithelial malignancies that can develop in any segment of the biliary tree. It is the second most common primary liver tumor, with an increasing incidence over the past decades, particularly in Western countries. CCAs exhibit heterogeneous clinical features, genotypes, and biological behaviors, depending on the anatomical location, growth pattern, and histological differentiation [98,99]. CCA can be categorized into three different forms, named intrahepatic, perihilar, and distal CCA, each of which are characterized by their own pathogenesis, risk factors, and clinical outcome [98,99]. In addition to an increasing incidence, the prognosis of CCA remains poor, with a median survival of less than 2 years and a survival rate of less than 10%. Due to the lack of early symptoms and specific biomarkers, CCA diagnosis is frequently reached at advanced stages, when treatment options are precluded. Surgical resection remains the main potentially curative treatment, even though this approach can only be performed in about 30% of patients, particularly those with intrahepatic CCA (iCCA). Unfortunately, the rate of recurrence after resection is still heavy, ranging from 50% to 60%, and survival at 5 years is less than 45% [100,101,102,103]. Another therapeutic option with a curative intent is represented by orthotopic liver transplantation following neoadjuvant chemotherapy. This procedure is becoming the standard of care for the treatment of localized iCCA, though it can only be offered to highly selected patients by a few specialized centers. Moreover, the procedure is contraindicated by the presence of comorbidities or the neoplastic involvement of regional lymph nodes. Following liver transplantation, disease-free survival ranges from 23% (for distal CCA) to 65% (for iCCA and hilar CCA), and thus depends on tumor localization [101,102,103]. Loco-regional treatments, such as transarterial chemoembolization or radioembolization, can also be taken into account for iCCA patients, but the results are limited [103]. Although CCA has been classically regarded as a highly resistant-to-chemotherapy tumor, little progress has been made over the last years. Combining gemcitabine and cisplatin has become the standard of care for patients with advanced CCA, showing an overall survival of 11.7 months, compared to 8.2 months in patients treated with gemcitabine alone [104]. In addition, several studies and clinical trials have been conducted aiming to tailor the most effective drug based on genetic signatures of the tumor [103,105]. Based on this, novel approaches have been devised, such as new small molecules to specifically target mutations or signal pathways detected in a single patient, or immunotherapy to inhibit one or multiple checkpoints, eventually in combination with chemotherapy or surgical resection [103,105]. However, data on immunotherapy in CCA are still limited, even if the anti-PD-1 monoclonal antibody pembrolizumab seems to be promising in patients with advanced CCA [106]. Mechanisms underpinning chemoresistance in CCA include escape from drug-induced apoptosis by cancer cells, mediated by autocrine and paracrine cues released in the tumor microenvironment [107,108]. Therefore, overcoming apoptosis resistance by inducing necroptosis in cancer cells may provide an attractive tool in the setting of CCA.

Although research on the role of necroptosis in CCA is at its dawn, a few important first steps have been made. A recent study on mice addressed how a hepatic microenvironment, created by two different methods of oncogenic delivery, could influence the type of hepatic tumor [109]. The authors showed that the same oncogenic drivers delivered to mature hepatocytes gave rise to iCCA when embedded in a necroptosis-dominated microenvironment, while an apoptotic milieu favored the development of HCC [40,109]. The necroptosis microenvironment was characterized by specific inflammatory cytokines (e.g., C-C motif ligand 6) secreted from immune cells, which were, in turn, activated by DAMPs released from necroptotically dying hepatocytes [18,40,59]. Transformed differentiated hepatocytes that were not surrounded by a necroptotic microenvironment gave rise to HCC or combined iCCA-HCC. Notably, transformed cholangiocytes could only degenerate into CCA and not HCC [40,109]. Further analysis found no role for stellate cells, immune cells, or overall death in modulating the hepatic microenvironment, which also seems to be independent of the oncogenic driver used [40,109]. Moreover, in the necroptotic microenvironment, the administration of necrostatin-1 (Nec-1) not only inhibited necroptosis, but also reduced cytokine release and shifted death from necroptosis to apoptosis, switching iCCA to HCC development [40,109]. Similar results were observed in hepatocyte-specific MLKL-KO mice [40,109]. Of note, the shift in tumor type occurred during the tumorigenesis phase, and not when the tumor was already developed [40,109]. The underlying molecular mechanisms that determine the preference of hepatocytes for apoptosis or necroptosis are still largely unknown, and whether or not the type of CD could also influence the tumor type in human chronic liver diseases has not yet been clarified [40,109]. In summary, Seehawer et al. provided an innovative insight into how the tumor microenvironment can be shaped by a specific CD type, eventually regulating lineage commitment in liver tumorigenesis [40]. These findings suggest that, during chronic liver diseases, a shift towards less fatal conditions with an improved prognosis could be possible, thus changing the natural course of disease progression [40,109].

Very few literature data regarding the prognostic role of necroptosis markers in CCA are currently available. Our group investigated this topic for the first time, in a series of 61 well-characterized and selected small and large bile duct iCCAs, by using immunostaining for RIPK3, RIPK1, and pMLKL. We found that all of these necroptosis-related proteins were highly expressed in the majority of cases, implying the presence and activation of the necroptotic cascade in iCCA. Moreover, their expression was found to be inversely correlated with the presence of iCCA negative prognostic factors, such as perineural invasion and nodal metastasis. Notably, we observed a significantly increased overall survival in patients with higher RIPK3 and RIPK1 expression. Furthermore, we described a direct relationship between RIPK3 expression and the number of intratumoral CD8+ T cells (only abstract available [110]). Therefore, overall, our preliminary data suggest a favorable role of necroptosis in iCCA.

## 6. Necroptosis-Based Therapies for Cholangiocarcinoma

Over the last years, several medications have been developed to modulate necroptosis, and numerous studies have provided the basis for moving some of these therapies toward clinical settings [2,18,31,54,55,59,72,111,112]. From what has been reported above, it is clear that RIPK1 and RIPK3 functions are essential for necroptotic cascade initiation and that it is possible to regulate necroptosis by acting on the functions of these molecules [4]. For example, Nec-1 is a well-investigated inhibitor of necroptosis that targets the catalytic and allosteric functions of RIPK1, thus preventing formation of the necrosome. Caspase-8 is able to block necroptosis by cleaving RIPK1 and RIPK3, and its activity can be modulated, both pharmaceutically and genetically (e.g., by deletion), to favor necroptosis [113]. At present, one of these drugs, an RIPK1 inhibitor, is in a phase 1 human clinical trial for the treatment of amyotrophic lateral sclerosis [112]. Moreover, some RIPK1 and RIPK3 inhibitors will likely be tested for chronic liver diseases very soon [59]. In the cancer setting, over the last few years, several methods have been studied to induce necroptosis as a putative therapeutic agent: radiotherapy, classical chemotherapeutic agents (such as 5-fluorouracil), kinase inhibitors (such as dorsomorphin), death receptor ligands (such as the CD95 ligand), oncolytic viruses, metal nanoparticles, Smac mimetics (RIPK1 activators), proteasome inhibitors, obatoclax (an inhibitor of anti-apoptotic Bcl-2 proteins), and polyinosinic:polycytidylic acid (PolyI:C; a TLR3 agonist) [6]. Combining necroptosis-inducing strategies with anti-apoptosis drugs allows apoptosis resistance to be easily bypassed. For instance, PolyI:C in combination with zVAD (a pan-caspase inhibitor) resulted in necroptotic CD in colon cancer, both in vivo and in vitro [33]. Concordantly, Smac mimetics combined with caspase-8 inhibitors have been shown to be effective in human acute myeloid leukemia cells and ovarian cancer cell lines and xenografts [114,115]. This suggests that inducing necroptosis could be used as a second-line treatment in cancer patients after the development of resistance to apoptosis [116,117,118]. Natural compounds such as shikonin (a component of a Chinese herbal medicine), staurosporine (an alkaloid extracted from the bacterium *Streptomyces staurosporeus*), neoalbaconol (a compound of a fungus), and bufalin (an ingredient of a Chinese medicine isolated from the skin and glands of toads) were also able to trigger necroptosis, with anti-tumoral outcomes [6,119,120,121]. Recent results of some clinical studies confirmed that necroptosis promotes natural or therapy-driven anti-cancer immunosurveillance in different neoplasms, including breast and ovarian carcinoma [3].

A widely adopted approach for killing cancer cells is to activate apoptosis, and evasion from this type of CD is considered a key step to therapeutic failure and chemotherapy resistance in CCA [122], as reported above. A switch to a non-apoptotic CD, such as necroptosis, can function as a backup system in apoptosis-resistant cells, so antitumor drugs inducing non-apoptotic CD are now considered a new approach for overcoming such a big obstacle in CCA treatment [0]. Only two studies have investigated the efficacy of different drugs in limiting CCA development by inducing necroptosis in CCA cell lines. Akara-amornthum et al. demonstrated that treatment with both TNFα and a Smac mimetic induced RIPK1/RIPK3/MLKL-dependent necroptosis when caspase was blocked, evidenced by an increased expression of RIPK3 and MLKL in CCA cell lines after treatment [122]. They also proved that the administration of a Smac mimetic could sensitize CCA cells to a low dose of standard chemotherapy with gemcitabine, by increasing TNFα mRNA levels and reversing gemcitabine-induced cell cycle arrest, leading to CD [122]. Xu et al. reported, for the first time, that matrine, an alkaloid isolated from traditional Chinese medicine *Sophora flavescens*, induced necroptosis in CCA cell lines, differing from its classical role of inducing apoptosis in many other types of cancer cells [123]. The authors showed that CCA cells under matrine treatment exhibited a typical necrosis-like morphology instead of apoptotic changes, and that these effects were greatly attenuated by Nec-1, but not by the apoptosis inhibitor zVAD [123]. A moderate expression of RIPK3 was observed in CCA cells and was required for matrine to induce necroptosis, which switched to apoptosis after endogenous R.

IPK3 knock down. Moreover, MLKL translocation from the cytoplasm to plasma membrane, as well as the increased production of ROS by RIPK3/MLKL, were critical for matrine to induce necroptosis. The authors also described a moderate increase in RIPK3 expression in most tissue samples from CCA patients, compared with adjacent normal tissues [0].

Overall, targeting necroptosis holds great promise for anti-cancer therapies, particularly in CCA patients, and ongoing efforts are focused on developing specific and stable pharmaceutical combinations.

## 7. Conclusions

CCA is an aggressive tumor with several clinical gaps that need to be filled. With limited treatment options at diagnosis, its management remains challenging. Therefore, it is crucial to develop effective therapeutic agents able to reduce the mortality of CCA patients.

Molecular insights into necroptosis mechanisms and their relevance in the pathobiology of liver diseases have been increased substantially over the last period. The necrosome components MLKL, RIPK1, and RIPK3, are critical regulators of necroptotic CD, with MLKL functioning as the executioner of necroptosis. Modulating necroptosis by regulating necrosome components might provide an interesting therapeutic tool.

Data on the role of necroptosis in CCA are scant but promising. Importantly, several questions need to be addressed in future research: What signal(s) triggers necroptosis in CCA cells during tumor development? Is the evasion of necroptosis a critical event for CCA development and progression? What are the regulatory mechanisms of necroptosis in CCA? An additional characterization of necroptosis, using specific biomarkers in human pathological samples, will be critical to unveiling the significance of necroptosis in CCA.

As new studies clarifying the mechanisms and the role of necroptosis in CCA are eagerly awaited in the near future, we believe that different therapeutic approaches should be explored and may hopefully result in innovative and effective treatments for CCA.

## Figures and Tables

**Table 1:** Pro- and anti-tumoral effects of necroptosis.

Pro-Tumoral Effects	Anti-Tumoral Effects
Necroptosis results in chronic inflammation and eventually tumorigenesis [32].	Cross-priming of cytotoxic CD8+ T cells enhances tumor clearance [33,34].
DAMP-induced inflammation activates the NF-κB pathway, inhibits apoptosis, and increases genomic instability [35,36].	RIPK3-induced activation of natural killer T cells promotes anti-tumor immunity [37].
Necroptosis of endothelial cells and extracellular matrix induces metastasis [29].	Necroptosis in apoptosis-resistant cancer cells inhibits carcinogenesis [38,39].
A necroptosis-dominant microenvironment induces lineage commitment towards intrahepatic CCA [40].	RIPK3 expression levels may predict the efficacy of cancer immunotherapy [41].
Necroptosis induces the production of molecules that promote an immune suppressive tumor microenvironment [30].	

## Abbreviations

AIH	autoimmune hepatitis
AIHI	acetaminophen-induced hepatic injury
ALT	alanine transaminase
ATP	adenosine triphosphate
BDL	bile duct ligation
CCA	cholangiocarcinoma
CD	cell death
cIAP	cellular inhibitor of apoptosis protein
DAMPs	damage-associated molecular patterns
FADD	Fas-associated protein with death domain
FLIP	FLICE-inhibitory protein
HCC	hepatocellular carcinoma
iCCA	intrahepatic CCA
IRI	ischemia/reperfusion injury
miR-21	microRNA-21
MLKL	mixed lineage kinase domain-like
NAFLD	non-alcoholic fatty liver disease
NASH	non-alcoholic steatohepatitis
NCCD	Nomenclature Committee on Cell Death
Nec-1	necrostatin-1
NF-κB	nuclear factor-kappa B
PolyI:C	polyinosinic:polycytidylic acid
RCD	regulated cell death
RIPK3	receptor-interacting serine/threonine-protein kinase 3
ROS	reactive oxygen species
Smac	second mitochondrial activator of caspases
TAK1	transforming growth factor β-activated kinase 1
TLRs	toll-like receptors
TNFα	tumor necrosis factor α
TNFR1	TNFα receptor 1
TRADD	TNFR1-associated death domain
